# Re-envisioning Technological Pedagogical Content Knowledge and Online Teaching Readiness of English for Foreign Language Pre-service Teachers in Language Teacher Education

**DOI:** 10.3389/fpsyg.2022.927835

**Published:** 2022-07-08

**Authors:** Karmila Rafiqah M. Rafiq, Melor Md Yunus, Susiati

**Affiliations:** ^1^Faculty of Education, Universiti Kebangsaan Malaysia, Bangi, Malaysia; ^2^Faculty of Teacher Training and Education, Universitas Muhammadiyah Surakarta, Surakarta, Indonesia

**Keywords:** EFL, online teaching and learning, pre-service teachers, teacher education, Technological Pedagogical Content Knowledge (TPACK)

## Abstract

The pandemic has brought online learning as the new norm in education, which invokes many issues, particularly in the quality of online education. The quality of teacher education is being questioned, particularly in imparting adequate Technological Pedagogical Content Knowledge (TPACK), which seems to be lacking in preparing teachers for sudden online learning. Though many studies were clustered around TPACK, there is still an unfilled gap in identifying whether the pre-service teachers are ready to teach online classes alongside their TPACK. Hence, this paper aims to determine (1) the level of TPACK and readiness and (2) the relationship between TPACK and readiness for English for Foreign Language (EFL) pre-service teachers in online learning during the pandemic. A survey was distributed to 197 EFL pre-service teachers with 35 items, in which the level of TPACK and readiness were analyzed descriptively with the mean score. The Pearson correlation examined the relationship between TPACK domains and readiness. The main findings showed that the level of TPACK and readiness of EFL pre-service teachers were high, particularly for Technological Knowledge (TK). However, the correlation results showed a weak positive correlation between TPACK domains and readiness. The highest correlation was between Technological Content Knowledge (TCK) and readiness, which is a new finding contributing to the body of knowledge. This paper implied that TPACK should also look into the online teaching readiness of pre-service teachers so that they are well equipped with the online pedagogical knowledge for effective teaching and learning.

## Introduction

The sudden shift of education from physical to online learning due to the pandemic has brought upon new worries and burdens around the globe, particularly in the quality of education. As stated in the fourth Sustainable Development Goal (SDG), everyone deserves quality and equal access to education, which seems to have become a widespread issue in certain lower-income countries (United Nations, 2021). Different countries face different types of problems, and one of them is the quality of teacher education. Though pre-service teachers spend a long time studying, their teaching intention is still unknown as they feel unprepared for real-life teaching (Astutik et al., 2022). This contributes to the debate on quality teacher education, particularly in Technological Pedagogical Content Knowledge (TPACK), which lacks training teachers to cope with online learning. TPACK is a framework consisting of a teachers’ knowledge. Teachers need to know TPACK to be effective educators, which means that teachers’ TPACK in teacher training programs is a guide for future teachers (Pazilah et al., 2021). Due to the COVID-19 pandemic, TPACK has become questionable, especially in online learning.

Online learning has become a norm, which education globally should embrace (Rafiq et al., 2021). However, the United Nations Educational Scientific and Cultural Organisation (UNESCO) reported that online education lacks quality because teachers are unprepared for online pedagogical knowledge. Only 43% from high-income countries and 56% from lower-middle and low-income countries received training in teaching online (UNESCO, 2020). These figures showed a worrying factor in ensuring the quality of online education, which comes back to the framework used in teacher education, TPACK (Pazilah et al., 2021).

Generally, a vast range of studies regarding TPACK has been carried out. Current research trends of TPACK reported on technology integration, mainly due to the current pandemic, which shifts education to an online setting. A study by Dalal et al. (2021) was carried out to identify the effectiveness of a course for pre-service teachers’ TPACK, and the findings revealed that the pre-service teachers improved more in the technological content knowledge (TCK), which relates to the pandemic situation of online learning. However, the same study reported that the pre-service teachers had difficulties adopting technology due to limited resources. This statement is supported by another survey of in-service teachers, which stated that TPACK correlated with the perceived usefulness and ease of using technology (Jang et al., 2021). These two studies implied that professional development courses should be geared toward teachers’ technological knowledge (TK).

Similarly, a study with 1144 teachers from around 64 countries categorized teachers into four profiles or categories known as low, medium, mixed and high. Though the medium and high teachers were optimistic and ready for online teaching with high technological support, the pedagogical support was reported to be low (Howard et al., 2021). One reason is the sudden change to online learning; hence not much support for professional development has been given to prepare the teachers. Valtonen et al. (2020) also mentioned that pedagogical knowledge played a vital role in ensuring the success of teaching and learning. This shows that regardless of the importance of technology in the current field, pedagogical knowledge still renders it crucial to be developed alongside other domains in TPACK. Additionally, some researchers reported that the level of TPACK domains is significant because it affects the teaching and learning quality (Naziri et al., 2019; Yanuarto et al., 2020; Fadzillah et al., 2021). This means that TPACK should be able to contribute to the quality of teacher education within its seven domains.

On another note of TPACK studies, multiple studies focused on teachers’ problems integrating technology in the online learning context. Many researchers reported similar findings that teachers, both pre-service and in-service, were facing difficulties in using technology to cater to online learning during this pandemic (Al-Abdullatif, 2019; Gyaase et al., 2019; Mare et al., 2019; Sarıçoban et al., 2019; Inpeng and Nomnian, 2020; Lie et al., 2020; Howard et al., 2021; Juanda et al., 2021; Masoumi, 2021; Syamdianita and Cahyono, 2021). These studies were carried out in different contexts regarding the readiness of teachers to integrate technology into online learning. Factors that hindered technology adoption include low readiness related to the TPACK domains. As mentioned in these studies, the low readiness factor was mainly associated with a lack of technological and pedagogical knowledge. Teachers, pre-service or in-service, face difficulties in balancing pedagogy and technology in online learning.

Nevertheless, one research reported contradicting findings carried out by Oh and Park (2018). It was mentioned that teachers’ technological competency and readiness were high. Though the results were relatively new, there is a need to note that the research was carried out in 2018, before the pandemic hit. Integrating technological tools in physical classrooms was a norm before COVID-19, yet the situation now requires new validation data. Researches in TPACK are also vastly carried out in various disciplines, such as sciences (Bonafini and Lee, 2021; Juanda et al., 2021; Ramnarain et al., 2021; Schmid et al., 2021), English language teaching (ELT) (Aşık et al., 2018; İşler and Yıldırım, 2018; Jwaifell et al., 2018; Lee and James, 2018; Sarıçoban et al., 2019; Singh and Kasim, 2019; Inpeng and Nomnian, 2020; Drajati et al., 2021; Syamdianita and Cahyono, 2021), preschool education (Masoumi, 2021), and special education (Oh and Park, 2018).

All these studies are clustered among TPACK and the technological competency of teachers. Interestingly, while many studies mentioned that teachers have low technological competency, Sarwa et al. (2020) reported that the North Sumatera teachers were ready to face 21st-century learning situations, though it was not mentioned which 21st-century learning skill was focused on. Another interesting discovery was disclosed by Jwaifell et al. (2018), where it was noted that female teachers’ readiness to use technology was higher than their male counterparts. Surprisingly, Al-Abdullatif (2019) reported similar findings, too, although the latter was not in the ELT field of studies. Recent research was carried out qualitatively to explore the factors relating to readiness to use technology in the classroom, based on the TPACK domains, which reported that different factors contributed to the lack of TK, such as resources and teacher training programs (Tiba and Condy, 2021). This shows that pre-service teachers need to be well equipped with TPACK to enhance their teaching ability in this pandemic.

Despite the tremendous amount of TPACK studies carried out in ELT, there is still a gap that is yet to be filled to identify the relationship of TPACK with technological readiness. Readiness in this study refers to the pre-service teachers’ intention to use technology in teaching despite having adequate TPACK. This is crucial for teacher training programs because it contributes to whether TPACK is adequate in imparting technological pedagogical and content knowledge for online teaching and learning, hence motivating this study to be carried out. The two objectives of this study are (1) to identify the level of TPACK domains of pre-service teachers and (2) to identify the relationship between TPACK domains and the technological readiness of pre-service teachers.

## Methods

### Research Design

This study employed the quantitative method. A survey design was used to identify the readiness of pre-service English for Foreign Language (EFL) teachers to employ technology for teaching and learning during COVID-19. The TPACK framework by Schmidt et al. (2009) is used alongside the Means Situation Analysis by Dudley-Evans and St John (1998). This Means Situation Analysis deals with pre-service teachers’ readiness to use technology to aid their online teaching and learning.

### Research Sample

A total of 197 pre-service teachers majoring in EFL from a university in Indonesia were chosen as respondents for this study. The sampling was selected through the purposive sampling method. They fit the category of pre-service teachers in year 2, majoring in EFL and currently enrolled in the Techniques to ELT course.

### Research Instrument

The cross-sectional survey method of data collection was employed, with 32 items. Twenty seven items for TPACK domains adapted from Schmidt et al. (2009), and five items for technological readiness adapted from Dudley-Evans and St John (1998). An expert checked the questionnaire in the related field to address the face and content validity. The TPACK domains from Schmidt et al. (2009) have reliability values between 0.75 and 0.95, which shows high reliability. The Dudley-Evans and St John (1998) means analysis construct has a Cronbach’s alpha value of 0.834.

### Data Analysis

The data was collected through an online platform, whereby all respondents were given a maximum of 30 min to answer the questionnaire as it is sufficient for them to answer 32 items. The researchers were present during the data collection in a virtual meeting space, which allowed the respondents to ask questions if they could not understand. All data collected were analyzed descriptively, representing the first objective of this study, which was to identify the level of TPACK and readiness of EFL pre-service teachers. The Pearson correlation analysis was used to condone the second objective to determine the relationship between TPACK domains and readiness to use technology. All data were analyzed with the Statistical Package for Social Sciences (SPSS) version 22.

## Results

### Research Objective 1: To Identify the Level of Technological Pedagogical Content Knowledge Domains of Pre-service Teachers

Referring to objective 1 of this study, TPACK domains and readiness were analyzed descriptively with the means for each domain. The mean interpretation followed the guidelines of interpretation adapted from Noor and Yunus (2017), as shown in Table 1.

**TABLE 1:** Mean interpretation.

Mean score	Interpretation
1.00–1.75	Strongly disagree (Very low)
1.76–2.50	Disagree (Low)
2.51–3.25	Agree (High)
3.26–4.00	Strongly agree (Very high)

The level of TPACK and technological readiness of EFL pre-service teachers are portrayed in Table 2.

**TABLE 2:** Technological Pedagogical Content Knowledge (TPACK) and technological readiness.

Construct	Abbreviation	Mean
Technological Knowledge	TK	2.97
Pedagogical Knowledge	PK	2.80
Content Knowledge	CK	2.74
Technological Pedagogical Knowledge	TPK	2.91
Technological Content Knowledge	TCK	2.88
Pedagogical Content Knowledge	PCK	2.77
Technological Pedagogical Content Knowledge	TPACK	2.84
Readiness	-	2.92

Based on Tables 1, 2, the mean showed that EFL pre-service teachers have high TK and TPK within the TPACK components, while their readiness to use technology is also high. Generally, looking into the mean score, it can be seen that EFL pre-service teachers have high TPACK knowledge, and they are aware of TPACK itself. Plus, they are generally ready to use technology in their classroom to cater to online learning during the pandemic.

### Research Objective 2: To Identify the Relationship Between Technological Pedagogical Content Knowledge Domains and the Technological Readiness of Pre-service Teachers

Referring to the second objective of this study, the Pearson correlation analysis was carried out to identify the relationship between TPACK and EFL pre-service teachers’ technological readiness. The findings are displayed in Table 3.

**TABLE 3:** Correlation between Technological Pedagogical Content Knowledge (TPACK) and readiness.

Constructs	TK	CK	PK	TCK	TPK	PCK	TPACK
Readiness	0.180*	0.208**	0.263**	0.384**	0.273**	0.285**	0.279**

*Indicates significant at level 0.05.

**Indicates significant at level 0.01.

As depicted in Table 3, generally, there is a positive correlation between TPACK and the readiness of EFL pre-service teachers to use technology to enhance language learning in this challenging time. For this study, the correlation interpretation by Profillidis and Botzoris (2019) is used as a reference, as shown in Table 4.

**TABLE 4:** Correlation interpretation.

Correlation	Interpretation
0	No correlation
0 < *r* < 0.3	Weak positive correlation
0.3 < *r* < 0.6	Moderate positive correlation
0.6 < *r* < 1	Strong positive correlation
1	Perfect positive correlation

The findings are interesting as TK is reported to have the weakest correlation with readiness to use technology in teaching (*r* = 0.180). However, TCK has a moderate positive correlation, which in this study is the highest correlation (*r* = 0.384). The correlation between technological readiness and other domains in TPACK is positively weak, with CK (*r* = 0.208), PK (*r* = 0.263), TPK (*r* = 0.273), PCK (*r* = 0.285), and TPACK (*r* = 0.279). Looking further into the findings, most TPACK domains fall in the weak positive correlation with readiness to use technology. Though they have a high level of TK as interpreted in the mean, it has a weak correlation with readiness.

## Discussion

This study aimed to cater to two objectives, which are (1) to identify the level of TPACK of EFL pre-service teachers and (2) to identify the relationship between TPACK domains and the technological readiness of EFL pre-service teachers. Referring to the first objective, the findings revealed that the EFL pre-service teachers involved in this study generally portrayed a high level of TPACK knowledge, with the highest being the TK domain. This is in line with previous studies (Mare et al., 2019; Sarıçoban et al., 2019; Singh and Kasim, 2019), which mentioned that, in general, pre-service teachers have sufficient knowledge of the TPACK domains, including the TK. Additionally, the technological readiness in this study had a similar mean to TK (mean = 2.97), which showed that the EFL pre-service teachers are ready to integrate technology in the classroom because of the high mean value. However, the notion of high TK and high technological readiness does not reflect that these EFL pre-service teachers effectively use technology in online learning. This statement is supported by various past studies, which reported that though the EFL pre-service teachers perceived their TK to be high, their ability to adopt technology effectively in the online learning context is low (Gyaase et al., 2019; Mare et al., 2019; Sarıçoban et al., 2019; Inpeng and Nomnian, 2020; Lie et al., 2020; Juanda et al., 2021; Syamdianita and Cahyono, 2021). One reason for this is the sudden shift in education from physical to online learning, which leaves a gap in the TPACK framework in terms of online pedagogy. This opens up opportunities for upcoming research to revise the framework in accordance with the current pandemic learning situation.

Next, looking into the second objective, findings showed a weak positive correlation between the TPACK domains and the technological readiness of EFL pre-service teachers. Although the findings showed a weak to moderate correlation, a positive relationship proves that TPACK domains somewhat correlate with technological readiness. The most significant finding is the correlation between TK with readiness, which showed the weakest correlation of all the other domains in TPACK. This is reflected by the fact depicted in other studies, which mentioned that even though the TK was high, the readiness of teachers to use technology effectively was still low (Gyaase et al., 2019; Mare et al., 2019; Lie et al., 2020; Syamdianita and Cahyono, 2021). With this fact in mind, TK does not correlate with the readiness of EFL pre-service teachers; instead, the domain portrays various alternatives for technology integration in the classroom. This is supported by Singh and Kasim (2019), who revealed that teachers could understand and master TPACK with sufficient TK to capture students’ attention in learning. From this, it can be deduced that the TK domain in TPACK is a domain that provides language teachers with proper technological skills to be used in the classroom.

Another novel finding from the correlation analysis was that the highest correlation was between the TCK domain and technological readiness. Though the correlation was moderate, it is the highest value for this study, which shows that TCK correlates to EFL pre-service teachers’ readiness to use technology. This finding is relatively new because previous studies reported Pedagogical Content Knowledge (PCK) (Valtonen et al., 2020) and Technological Pedagogical Knowledge (TPK) (Dalal et al., 2021) as the most vital domain in ensuring the success of TPACK in language teacher education. Although previous studies were carried out recently, during the pandemic, it can be seen that pedagogical knowledge played an important role in teacher education programs, which depicts that all teachers should have proper pedagogy to teach effectively. Regardless, TCK relates to the EFL pre-service teachers the most in this study, which brings a new light to look into the TPACK domains, particularly in catering to the needs of education in this COVID-19 era. One possible reason for TCK emerging as the most influential domain regarding readiness is that education is online. Teachers teach in a virtual space, thus bringing more importance to content and technology mastery. From this study also, it can be seen that TK has the weakest correlation value. Still, TCK has the highest correlation value, which enlightens the body of knowledge, particularly in the TPACK framework. This opens up the opportunity for future research to look into how TCK could enhance TPACK in the context of online education.

## Conclusion

This study aims to identify the level of TPACK and readiness to use the technology of EFL pre-service teachers during the pandemic. The main findings depicted that EFL pre-service teachers have high TPACK and readiness to use technology in the classroom, showing that they are mentally prepared to teach in an online setting, including the TPACK domains necessary for teaching. Another finding showed a positive correlation between the TPACK domains and readiness to use technology in the classroom during the pandemic. Though the correlation is generally weak to moderate, the positive correlation indicates that TPACK knowledge relates to EFL pre-service teachers’ readiness to use technology in online teaching and learning. This study contributes to the gap in identifying EFL pre-service teachers’ TPACK level and technological readiness in this pandemic era of language learning.

Stakeholders, curriculum planners, pre-service teachers, and teacher training programs will benefit from this study in rectifying and refining the contents in teacher training programs, which could be enhanced during this unexpected pandemic. The findings from this study implied that TPACK should also look into the online teaching readiness of pre-service teachers so that they are well equipped with the online pedagogical knowledge for effective teaching and learning. The limitations of this study are in terms of the samples used and the cross-sectional study design. The samples are limited to only year 2 EFL pre-service teachers in a university in Indonesia. The gender factor is not included in this study due to the huge imbalance of male and female respondents. Also, the cross-sectional survey design is used due to the time constraint. Future studies can address these limitations by comparing the TPACK and pre-service teachers’ readiness in terms of gender or year of studies or by carrying out a longitudinal study, which could provide better insights into the current TPACK model regarding readiness to use technology in online classrooms.

## Data Availability Statement

The original contributions presented in this study are included in the article/supplementary material, further inquiries can be directed to the corresponding author.

## Author Contributions

KRMR and MMY conceived the study and participated in its design and coordination. All authors collected field data, entered study data, assisted in data analysis and interpretation of study results, performed final analyses, co-drafted the manuscript, read, revised, and approved the final manuscript.

## Conflict of Interest

The authors declare that the research was conducted in the absence of any commercial or financial relationships that could be construed as a potential conflict of interest.

## Publisher’s Note

All claims expressed in this article are solely those of the authors and do not necessarily represent those of their affiliated organizations, or those of the publisher, the editors and the reviewers. Any product that may be evaluated in this article, or claim that may be made by its manufacturer, is not guaranteed or endorsed by the publisher.
